# Quality of transitional care of children with chronic diseases: a cross-sectional study

**DOI:** 10.1590/1980-220X-REEUSP-2021-0535

**Published:** 2022-04-08

**Authors:** Caroline Cechinel-Peiter, Gabriela Marcellino de Melo Lanzoni, Ana Lúcia Schaefer Ferreira de Mello, Aline Marques Acosta, Juliana Coelho Pina, Selma Regina de Andrade, Nelly Donszelmann Oelke, José Luís Guedes dos Santos

**Affiliations:** 1 Universidade Federal de Santa Catarina, Florianópolis, SC, Brazil.; 2 Universidade Federal do Rio Grande do Sul, Porto Alegre, RS, Brazil.; 3 The University of British Columbia, Campus Okanagan, Kelowna, British Columbia, Canadá.

**Keywords:** Child, Chronic Disease, Continuity of Patient Care, Nursing Care, Patient Discharge, Transitional Care, Criança; Doença Crônica, Continuidade da Assistência ao Paciente, Cuidados de Enfermagem, Alta do Paciente, Cuidado Transicional, Niño, Enfermedad Crónica, Continuidad de la Atención al Paciente, Atención de Enfermería, Alta del Paciente, Cuidado de Transición

## Abstract

**Objective:**

To analyze which factors may be associated with the quality-of-care transition of children with chronic diseases from the hospital to their home.

**Method:**

A cross-sectional, quantitative study, carried out in two hospitals in Southern Brazil, from February to September 2019. Participants included 167 family members of children with chronic disease. Data collection took place through a demographic questionnaire, and the use of the Brazilian version of the Care Transitions Measure (CTM-15).

**Results:**

The average score for the quality of care transition was 90.1 (sd = 19.5) (0–100). Factor 1, “Health management preparation”, was the one with the highest self-perceived average, 92.3 (sd = 11.6), while Factor 4, “Care plan”, had the lowest average, 86.3 (sd = 21.3). The quality of care transition was higher for patients living in municipalities belonging to health regions other than the hospital’s.

**Conclusion:**

The quality of care transition for children with chronic diseases, perceived by the children’s family members, in the discharge process from the hospital to home, was considered high. Living in a health region other than the hospital’s region was associated with better perception of the quality of care transition.

## RESUMO

**Objetivo:**

Analisar que fatores podem estar associados à qualidade da transição do cuidado do hospital para o domicílio de crianças com doenças crônicas.

**Método:**

Estudo transversal, quantitativo, realizado em dois hospitais do Sul do Brasil, de fevereiro a setembro de 2019. Participaram 167 familiares de crianças com doença crônica. A coleta de dados ocorreu por meio de um instrumento sócio-demográfico e da da versão brasileira do Care Transitions Measure (CTM-15).

**Resultados:**

A pontuação média para a qualidade da transição de cuidados foi de 90,1 (dp = 19,5) (0–100). O Fator 1, “Preparação para o autogerenciamento”, foi o fator com maior média autopercebida, 92,3 (dp = 11,6), enquanto o Fator 4, “Plano de cuidado”, teve a menor média, 86,3 (dp = 21,3). A qualidade da transição de cuidado foi maior para os pacientes residentes em municípios que não pertenciam à mesma região de saúde dos hospitais.

**Conclusão:**

A qualidade da transição do cuidado de criança com doenças crônicas, percebida pelos familiares, no processo de alta hospitalar para o domicílio, foi considerada alta. Morar em outra região de saúde que não aquela do hospital associou-se a uma melhor percepção da qualidade da transição do cuidado.

## RESUMEN

**Objetivo:**

Analizar qué factores pueden estar asociados a la transición de la calidad del cuidado de niños con enfermedades crónicas del hospital para el domicilio.

**Método:**

Estudio transversal, cuantitativo, realizado en dos hospitales del Sur de Brasil, de febrero a septiembre de 2019. Participaron 167 familiares de niños con enfermedad crónica. La recolección de datos ocurrió a través de un cuestionario demográfico y de la utilización de la versión brasileña del *Care Transitions Measure* (CTM-15).

**Resultados:**

La puntuación promedia para la calidad de la transición de cuidados fue de 90,1 (dp = 19,5) (0–100). El Factor 1, “Preparación del manejo de la salud”, fue el factor con promedio auto percibido más alto, 92,3 (dp = 11,6), mientras el Factor 4, “Plan de cuidado”, tuvo el promedio más bajo, 86,3 (dp = 21,3). La calidad de transición de cuidados fue más alta para los pacientes que viven en municipios ubicados en regiones de salud que no la del hospital.

**Conclusión:**

La calidad de la transición del cuidado al niño con enfermedades crónicas, percibida por sus familiares en el proceso de alta hospitalaria al domicilio, fue considerada alta. Vivir en otra región de salud que no aquella del hospital tuvo relación con una mejor percepción de la calidad de la transición de cuidados.

## INTRODUCTION

Care transitions comprise actions that enable the coordination and continuity of care during the patient’s transfer between health services^(1)^. The concept of care transition covers a wide range of meanings that converge to a systematic care process focused on the patient, also involving family caregivers and members of the multidisciplinary team responsible for their care^(2)^.

The moment of transition from the hospital setting to home denotes vulnerability for continuity of care, since it depends on several factors, such as patient’s health needs and degree of dependence, the support network, and access to other services in the health care network, requiring coordination and communication between different professionals and points of care^(3,4)^.

Among the outcomes associated with high-quality transitions from hospital to home are improved self-care, quality of life, patient satisfaction, and continuity of care. Higher levels of quality in care transitions are related to decreased visits to hospital emergency services and increased adherence to medications as recommended^(5)^. Moreover, there is an improved cost- benefit ratio for acute care, as it contributes to the decrease of readmission rates and decreased length of stay^(2,6)^.

Patients with chronic diseases are particularly more vulnerable to fragmentation of healthcare, especially in the moment of hospital discharge. These patients require special attention from professionals and managers to address transitional care from hospital to home^(7)^. In Brazil, concerning child health care, 9.1% of children up to four years old and 9.7% of those from five to 13 years old have some chronic diseases^(8)^. The increased prevalence of chronic diseases in childhood requires a new organization of health systems, with integration of services for adequate management of increasingly prevalent chronic diseases^(7)^.

In this perspective, the Brazilian health system has mechanisms to facilitate comprehensive health care, such as Out- of-Home Treatment Program, which aims to ensure low-scale health treatment, beyond the patient’s municipality; Health Regulation, which seeks to optimize health resources based on the needs of the user population; and logistic systems, which seek integration and communication between the services in the Health Care Network. However, the consolidation of referral and counter-referral mechanisms is still incipient, making the integration of the health system still fragile^(8)^.

Due to the flaws in the integration of a child’s health care network of services and providers, there is a legitimate concern about how prepared health services and providers are to fully assist children and their families in the home setting^(9)^. These issues emphasize the need for investment in care transition programs, to strengthen continuity of care after discharge.

Transitional care strategies depend directly on the nurse. The qualified performance of nurses is essential for safe and quality transitions, in addition to providing visibility and appreciation to the professional^(10)^. Although care transitions are related to better outcomes for children with chronic diseases^(11)^, there is an important gap in the literature concerning the in-depth understanding of these individuals’ care transition^(12)^.

A better understanding of factors associated with the transitional care can help nurses, managers, and other health professionals to develop and apply strategies that qualify this process, impacting in better continuity of care and health quality over time. Thus, the research question is: Which factors may be associated with the quality of care transitions of children with chronic diseases from the hospital to home?

This study aimed to analyze which factors may be associated with the quality of care transitions of children with chronic diseases from the hospital to home.

## METHOD

### Design of Study

Cross-sectional, quantitative study, which complied with the STROBE checklist for cross-sectional studies.

### Population

Participants included family members responsible for children with chronic disease, who were discharged home.

### Local

The study was carried out in pediatric inpatient units in two large public hospitals in southern Brazil. Both institutions are important reference to high complexity for the pediatric care in the Health Region.

### Selection Criteria

Family members legally responsible for children up to nine years of age with previous diagnosis of chronic disease and who were discharged from hospital to home were included. To define chronic disease, a compilation of the highest prevalence in hospitalized children in Brazil was adopted^(13)^. We excluded children under 29 days of age, patients with a referral to other units or institutions, those who, at the time of telephone contact, had died or had been readmitted, and those caregivers under 18 years old.

Initially, 395 family members of patients were approached during hospitalization, of which 262 had at least one chronic disease, based on the inclusion criteria. Of these, 55 were older than nine years or less than 29 days, 35 did not answer our telephone calls, two were transferred to another institution, one child died between the invitation to participate in the research and the telephone call, one was readmitted, and one declined to continue in the study. Thus, 167 participants were included.

### Sample Definition

Sample calculation was performed with the aid of software Winpepi, version 11.65, considering a 95% confidence level, a margin of error of 4 points, a mean of 74.7, and a standard deviation of 17.1 points^(10)^. We considered an 80% power and a significance level of 5%, adding 5% for possible losses and refusals. The minimum sample size was 156 participants.

### Data Collection

Data collection was carried out from February to September 2019. A demographic questionnaire and the Brazilian version of the Care Transitions Measure instrument (CTM-15) were used^(14)^.

The dependent variable Quality of care transition was obtained by applying the CTM-15, composed of 15 items, with response options on a 4-point Likert scale (“Strongly disagree”, “Disagree”, “Agree”, “Strongly agree”, “I don’t know/I don’t remember/It does not apply”). Thus, the higher the score, the higher the quality of care transition. The instrument’s items are organized into four factors: 1) “Health management preparation” (4, 5, 6, 8, 9, 10 and 11); 2) “Medication understanding” (13, 14 and 15); 3) “Important preferences” (1, 2 and 3); and 4) “Care plan” (7 and 12)^(14)^.

The independent, categorical variables of the study were the respondent’s relationship with the child; the child’s sex and the municipality of residence; ICD 10 code for chronic disease; and the number of hospitalizations in the last 12 months. For the continuous variable, we used the child’s age (full years).

The invitation to participate in the research was made by the researcher through person-to-person contact during the hospitalization period. Upon consent of the child’s family members, the demographic questionnaire was completed.

The CTM-15 was completed via telephone with the same child’s family members, from seven to 30 days after hospital discharge, as recommended by the instrument’s authors^(14)^.

### Data Analysis

Data were entered and organized in an Excel spreadsheet and processed using the Statistical Package for the Social Science (SPSS) version 20 software.

Data were analyzed using descriptive and inferential statistics. CTM-15 final score was calculated using a formula with the averages of the scores found, converting them on a linear scale from zero to 100 points. As recommended by the instrument’s authors, the items answered with the option “I don’t know/ I don’t remember/It does not apply” were not counted in the average calculation of the final score^(14)^.

Student t-test, Analysis of Variance and Spearman Correlation tests were applied. The level of significance was set at 95% (p < 0.05).

### Ethical Aspects

The study followed the recommendations of Brazilian Resolution No. 466/2012, and was approved by the Human Research Ethics Committee of Universidade Federal de Santa Catarina, Brazil, under protocol No. 3.063.726 in December 2018. All participants signed the Informed Consent Form.

## RESULTS

The total of 167 participants were included in the research. The family members in attendance in hospitals of children with chronic diseases were, mostly, mothers (130;77.8%). Among the children discharged, most were male (95;56.9%); children’s mean age was 3.7 years (sd = 2.9). The majority came from municipalities in the same health region as the institutions (113;67.7%). Respiratory diseases (60;35.9%) and malformations (50;29.9%) were the predominant chronic diseases. Of the total number of children, 103 (61.7%) were hospitalized for the first time in the last 12 months (Table 1).

**Table 1.** Characteristics of patients included in the study (n = 167) – Florianópolis, SC, Brazil, 2019.

Variables	n	%
**Family members**		
Mother	130	77.8
Father	26	15.6
Other (Grandmother, aunt or stepmother)	11	6.6
**Sex**		
Male	95	56.9
Female	72	43.1
**Age**		
Children under 1 year	30	18.0
1 to 2 years	22	13.2
2 to 5 years	55	32.9
5 to 9 years	60	35.9
**Municipality of Residence**		
Same health region as hospitals	113	67.7
Other health region	54	32.3
**ICD 10 codes for chronic disease**		
Respiratory system diseases	60	35.9
Congenital malformations, deformities and chromosomal abnormalities	50	29.9
Neoplasms	15	9.0
Diseases of the circulatory system	11	6.6
Nervous system diseases	9	5.4
Endocrine, nutritional, and metabolic diseases	7	4.2
Diseases of the digestive system	5	3.0
Diseases of the genitourinary system	5	3.0
Mental and behavioral disorders	3	1.8
Diseases of the blood and blood-forming organs and some immune disorders	2	1.2
**Number of hospitalizations in the last 12 months**		
1	103	61.7
2–3	49	29.3
4–5	11	6.6
6 or more	4	2.4

Source: research data.

The mean total score of CTM-15 was 90.1 (sd = 19.5). In the assessment of specific instrument items, the highest scores corresponded to item 9, “Understand things I was responsible for” (95.4), and to item 14, “Understand how to take medications” (96.1). The item with the lowest score was item 15, “Understand side effects of medications.” (75.8) (Table 2).

**Table 2.** Average and standard deviation of the scores obtained, according to the items of CTM-15 instrument – Florianópolis, SC, Brazil, 2019.

	Item	Factor	Average	Standard deviation
14	Understand how to take medications	2	96.1	±13.2
9	Understand things I was responsible for	1	95.4	±11.6
5	Understand how to manage health	1	93.5	±17.2
8	Understand what makes me better or worse	1	93.3	±14.8
13	Understand purpose of medications	2	93.0	±19.0
6	Understand warning signs and symptoms	1	92.7	±17.3
4	Had information needed for self-care	1	92.5	±17.8
10	Feel confident that you know what to do	1	91.1	±18.1
11	Confident I knew what to do	1	91.1	±17.3
1	Agreed health goals and means	3	89.0	±18.1
2	Preferences for deciding health needs	3	87.6	±24.0
3	Preferences for deciding where needs are	3	87.1	±22.6
12	Had written list of appointments and tests	4	86.8	±25.5
7	Had written care plan	4	85.7	±26.7
15	Understand side effects of medications	2	75.8	±35.3

Source: research data.

Regarding the four factors of the instrument, Factor 1, “Health management preparation”, was the one with the highest mean, 92.3 (sd = 11.6), while Factor 4, “Care plan”, presented the lowest mean, 86.3 (sd = 21.3). Factors 2, “Medication understanding”, and 3, “Important preferences”, were 88.3 (sd = 17.5) and 87.7 (sd = 17.1), respectively.

Regarding the study’s independent variables, we found that patients from municipalities that did not belong to the same Health Region as hospital institutions had a higher score in the quality index of care transition than those in the same health regions. There was no statistically significant difference in these associations and correlations between the CTM-15 linear scale and the other independent variables (Table 3).

**Table 3.** Association of the CTM-15 score with the study variables – Florianópolis, SC, Brazil, 2019.

Variables	Average	Standard deviation	P value
**Family members**			
Mother	**89.6**	±11.9	0.282**
Father	**92.1**	±10.2	
Other (Grandmother, aunt or stepmother)	**91.0**	±12.7	
**Sex**			
Male	**91.3**	±**9.6**	0.196*
Female	**88.6**	±**13.9**	
**Age**			
Complete years	+0.105***		0.178***
**Municipality of residence**			
Same health region of hospitals	**88.9**	±12.3	0.048*
Other health region	**92.7**	±10.0	
**ICD 10 codes for chronic disease**			
Respiratory system diseases	**88.7**	±**12.6**	
Congenital malformations, deformities and chromosomal abnormalities	**90.6**	±**11.6**	
Neoplasms	**92.4**	±**7.8**	
Diseases of the circulatory system	**94.9**	±**4.3**	0.812**
Nervous system diseases	**90.6**	±**13.3**	
Endocrine, nutritional and metabolic diseases	**90.5**	±**13.4**	
Diseases of the digestive system	**91.5**	±**3.2**	
Diseases of the genitourinary system	**87.6**	±**16.5**	
Mental and behavioral disorders	**88.7**	±**14.2**	
Diseases of the blood and blood-forming organs and some immune disorders	**80.0**	±**28.3**	
**Number of hospitalizations in the last 12 months**			
1	90.4	11.5	0.209**
2–3	89.2	±12.8	
4–5	92.7	±5.7	
6 or more	83.2	±15.9	

Source: research data. *Student t-test; **Analysis of variance; ***Spearman correlation.

## DISCUSSION

This research sought to analyze the quality of care transitions from hospital to home, from the perspective of family members of children with chronic diseases. These results contribute to guiding professionals and researchers in actions to improve care transition process^(1)^, especially in the context of hospital care for children.

The overall average of the CTM-15 linear scale, in this research, was 90.1. Although the instrument does not have a predefined cutoff point, this average can be considered high. Studies previously developed in Brazil, using the instrument in adult patients, presented averages ranging from 69.5 to 79.0^(10–14)^. These results are close to those found in studies carried out in Sweden, United States and Japan, where the average was 65.8, 78.5 and 66.3, respectively^(1,5,15)^.

Another study that applied the summarized version of the CTM in assessing the transition of childcare, averaged 83.7^(16)^. Care at home requires an understanding of the new reality for the children with chronic diseases and their family. These may involve complex changes in the family’s adaptation due to the limitations of the chronic diseases and necessary adjustments for its management^(17)^. Moreover, pediatric care is family-centered, which may influence professionals’ concern with the quality of care transition, to improve the continuity of care in the home context. These may explain some of the differences in the results of CTM-15 average score compared to those found in adults.

About the averages per item of the instrument, items 9 “Understand things I was responsible for” and 14 “Understand how to take medications” showed the highest scores. Based on the child’s life stage, the caregivers would intrinsically understand their responsibility to care for the child. On the other hand, while in the hospital, families, especially mothers, could be seen as a person who would provide care to the child, often being assigned responsibilities that would belong to the care team, making them feel responsible for the care, beyond their skills^(18)^.

Although item 14, “Understand how to take medications”, showed one of the highest averages, the worst assessment for the quality of care transition in this study was in item 15 “Understand side effects of medications”. This aligns with another study, in which 57% of patients did not clearly understand the possible side effects of each of their medications when leaving the hospital^(19)^. Likewise, in another study that also used CTM in the assessment of care transition of elderly patients, this item also had the worst average^(20)^. Insufficient information on side effects is associated with low adherence to therapy and a higher risk of an adverse reaction, leading to worsening of a preventable condition^(19)^.

The instrument’s Factor 1, “Health management preparation”, presented the highest average. This could be because of the emphasis on the need for health education, with its understanding and incorporation into practice by professionals. Health education for the family improves autonomy and co-responsibility in decision-making and allows family-centered care, which is crucial for successful care at home^(17,21)^.

Health education is one of the main strategies in care transition and involves several aspects of the patient and family’s life^(22)^. This is an important concern of nurses in the transitional care, focusing in the reality of each family and considering the changes and adaptations when facing the health condition^(17,22)^. In this context, health education actions stand out, such as the handout of folders, use of simple and appropriate language and confirmation that the message received was understood by the patient and caregiver^(22)^.

Factor 4, “Care plan”, had the lowest average, suggesting that the availability of formal guiding documents still requires work, which may be related to the fact that the hospitals from the study do not have formal care transition programs implemented, and do not have institutional strategies guiding transition actions. The guidelines for hospital discharge are often scarce, carried out in a generalized manner, without considering the specific needs of the patient, and without previous planning, being limited to the moment when the patient leaves the hospital^(23)^.

Care plan is one of the main interventions at the time of hospital discharge, leading to clear, objective and effective communication among the professional, the patient and the family^(24,25)^. Despite the significance of organizing care guidelines in a systematic plan, the guidance on care at home takes place primarily verbally and spontaneously. Furthermore, in many cases, these guidelines are complex, and should involve aspects such as drug therapy, prevention of exacerbations, food, and general care^(17,25)^, emphasizing the need for a formalized care plan.

The contribution of nurses in the transitional care shall be highlighted, as this professional plays an active role in transition strategies, especially concerning the identification of patient needs after discharge, health education, preparation for self-management of the disease at home, and communication between the multiprofessional team. In this way, the nurse’s performance is essential for a quality transition from the hospital to home^(26)^.

There was an association between the quality of care transition and the municipality where the child lives. Hospitalization increases parents’ confidence in care and safety for their child in the face of complications. Therefore, discharge represents an interruption of professional support and the end of care previously shared in the hospital environment^(17)^. Due to the parents’ feeling of insecurity after hospital discharge, health professionals may think that patients and families living in municipalities farther from the hospital have limited access to the hospital and therefore should receive greater attention on care transition. Thus, the concern of professionals regarding care transition would be greater in patients identified as at risk for increased fragmentation of care, justifying the difference between the perceived quality of the transition, higher on the municipalities closer the hospitals.

Literature associates repeated admissions and low levels of transitional care, considering that bad transitions can result in new admissions, and professionals may assume that frequently admitted patients are aware of their needs. Nonetheless, this study did not find any significant results in the association of the number of admissions in the last year, and the quality of transitional care^(22)^.

One limitation of the study is that the results of care transition evaluation through the CTM-15 may have been influenced by the feeling of gratitude that families have for the health service. Due to the assistance received at the time of the child’s fragility, family members may feel obliged to give priority to affirmative responses, seeking to answer what they think the interviewers would like to hear.

## CONCLUSION

The quality of care transitions for children with chronic diseases, self-reported by their parents and evidenced in this study, was highly valuable, highlighting the training for Health management preparation and the understanding of the use of medicines. There was a weak point concerning the care plan and in understanding the drugs’ side effects. Patients living in municipalities belonging to health regions other than the one where the participating hospitals were located had a higher perceived quality of their care transition.

There is a need to further understand the mechanisms involved in the quality of care transitions, especially in child healthcare, considering the specificities of this population. However, few studies using CTM-15 in childcare transition assessment have been found, which highlight the originality of this study. Thus, this study can help nurses, managers and policy makers to understand the quality of care transitions concerning child healthcare, especially considering the gap on the literature about child care transition.

Other studies are required to improve the understanding of care transitions in children, to fill the gaps regarding aspects that affect this process and influence the quality of life and health of the patient and family, in their home environment. This will facilitate expanding evidence for transition programs to be adopted.

## ASSOCIATE EDITOR

Ivone Evangelista Cabral
